# Oligonucleotides Modified With Transplatin Derivatives: Fast and Efficient Metalloribozymes

**DOI:** 10.1155/MBD.2001.39

**Published:** 2001

**Authors:** Rozenn Dalbiès-Tran, Marc Leng, Marc Boudvillain

**Affiliations:** 1 Centre de Biophysique Moléculaire CNRS rue Charles Sadron Orléans cedex 2 F-45071 France; 2 Physiologie de la Reproduction et du Comportement INRA Nouzilly F-37380 France

## Abstract

When an oligonucleotide containing a 1,3-(G,G)-transplatin cross-link at a GNG site (N represents a C, T, A or U residue) is paired with its complementary strand, the intrastrand adduct rearranges into an interstrand cross-link, resulting in the covalent attachment of both strands. Here, we have studied the influence of the inert ligands of the platinum(II) complex and of the nucleotide residues in the vicinity of the adduct on the rearrangement reaction. Dramatic effects on the linkage isomerization rate could be 37℃. The results are analyzed in relation with the mechanism of rearrangement of the 1,3-intrastrand adducts into interstrand cross-links. The relevance of platinated oligonucleotides as potent and specific drugs is discussed.


### OLIGONUCLEOTIDES MODIFIED WITH TRANSPLATIN DERIVATIVES: FAST AND EFFICIENT METALLORIBOZYMES

Rozenn Dalbiès-Tran[1,2], Marc Leng[1] and Marc Boudvillain*[1]

[1] Centre de Biophysique Moléculaire, CNRS, rue Charles Sadron, F-45071 Orléans cedex 2, France

[2] Physiologie de la Reproduction et du Comportement, INRA, F-37380 Nouzilly, France (present address)

<boudvill@cnrs-orleans.fr>

### ABSTRACT

When an oligonucleotide containing a 1,3-(G,G)-transplatin cross-link at a GNG site (N represents a C, T, A or U residue) is paired with its complementary strand, the intrastrand adduct rearranges into an interstrand cross-link, resulting in the covalent attachment of both strands. Here, we have studied theinfluence of the inert ligands of the platinum(II) complex and of the nucleotide residues in the vicinity of the adduct on the rearrangement reaction. Dramatic effects on the linkage isomerization rate could be observed with half-lives of the intrastrand adducts ranging from a few minutes to over 50 hours at 37°C. The results are analyzed in relation with the mechanism of rearrangement of the 1,3-intrastrand adducts into interstrand cross-links. The relevance of platinated oligonucleotides as potent and specific drugs is discussed.

### INTRODUCTION

Many enzymes rely on metal cofactors to perform catalysis. It is the case for protein enzymes as well as DNA and RNA enzymes, the so-called ribozymes (1, 2). Several natural ribozymes (e.g. group I or group II introns) are not true enzymes since they are chemically modified during the very reactions they trigger. However, it is often possible to design *trans*-acting molecular systems derived from these natural ribozymes, in which one component acts as atrue enzyme, not altered by the reaction and capable to catalyze multiple rounds of the same reaction. Ribozymes have a great potential in biotechnology and medecine and new catalytic RNA or DNA molecules have been discovered by *in vitro* selection methods. Most of these synthetic ribozymes utilize metal cofactors such as $Mg^{2+}$, $Ca^{2+}$ or $Pb^{2+}$ (3).

The reaction between *trans*-diamminedichloroplatinum(II) (transplatin) (Figure 1) and single-stranded oligonucleotides containing a GNG sequence yields *trans*-{$Pt(NH_3)_2$[(GNG)-G*N*7,G*N*7]} intrastrand cross-links (1,3-(G,G)-intrastrand cross-links). These are usually stable within single-stranded oligonucleotides, with the exception of adducts formed at CGNG sites (4, 5). By contrast, instability appears tobe the general rule within the duplexes formed by the platinated oligonucleotides and their complementary DNA or RNA strands: 1,3-(G,G)-transplatin intrastrand cross-links rearrange into interstrand cross-links (6-8). In this reaction, by analogy with natural ribozymes, the nucleic acid double helix may be considered as a catalytic molecule (9). It should then be possible to split the platinated duplex into several individual components among which one *trans*-acting molecule is a true enzyme (for a hint at what could be done, see ref. (10)). Remarkably, this would represent the first example of a ribozyme in which the metal is not merely a cofactor but is in fact the substrate which undergoes the chemical reaction. In the present study, we have focused our attention on the molecular environnement of the platinum(II) moiety. We have characterized the influence on the rearrangement kinetics of its inert ligands as well as the role of the oligonucleotide sequence in its vicinity. Collectively, the results suggest that chemical bond strain and proper orbital steering, two factors often essential for enzymatic reactions, are important in the rearrangement reaction. In addition, the results also provide valuable information to develop further the use of platinated oligonucleotides as specific molecular tools in biotechnology and medecine (8, 11).

### MATERIALS AND METHODS

*Materials.* Oligodeoxyribonucleotides and oligo(2'-O-R-ribonucleotide)s from Eurogentec (Belgium) were purified by strong anion exchange chromatography, as described (5,6). The purified oligoribonucleotides were from Genset (France). T4 polynucleotide kinase was purchased from New England Biolabs (U.K.), [$^{32}P$]-$\gamma$ATP from Amersham Pharmacia Biotech, transplatin from Johnson-Mattey (U.K.), while platinum(II)

derivatives (1) to (4) (Figure 1) were kindly provided by Pr. Bernhard Lippert (University of Dortmund, Germany). All other chemicals were from Merck.

*Platination of the oligonucleotides.* Oligonucleotides containing a single 1,3-(G,G)-intrastrand cross-link were prepared and purified as described previously (6,8). Identification of the cross-linked residues was performed as described (6,11).

*Kinetics of the rearrangement reaction.* The platinated oligonucleotides (2 μM) were mixed with one equivalent of their complementary strands (a fraction of which was 5'-end labeled with [$^{32}$P]-γATP and polynucleotide kinase) and were incubated at 37°C in 150 mM $NaClO_4$, 5mM phosphate buffer and 0.2mM EDTA, pH 7.5 (6-8). At various times, aliquots were removed and analyzed by electrophoresis on a denaturing 24% polyacrylamide gel. Radioactive species were detected and quantitated using the Molecular Dynamics Phosphorimager/ImageQuant system. The half-lives of 1,3-intrastrand cross-links were determined based on 2 to 3 independent experiments as described (6). The mean standard error was less than 20%.

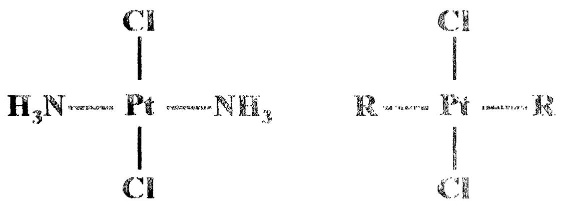

(1): R = $CH_3NH_2$
(2): R = $(CH_3)_2NH$
(3): R = $CH_3CH_2NH$
(4): R = $HO\text{-}CH_2CH_2NH_2$

**Figure 1:** formulas of transplatin (left) and of the other platinum(II) complexes used in this work.

### RESULTS

Until recently, bifunctional cross-links of platinum(II) within duplex DNA were usually considered thermodynamically and kineticallystable. Our laboratory demonstrated that 1,3-(G,G)-intrastrand cross-links formed by transplatin in single-stranded oligonucleotides spontaneouslyevolve into interstrand cross-links upon pairing with the complementary DNA or RNA strands (6). These later species are stable over long periods of incubation (6,8). Since this first observation, we have prepared and studied many such platinated oligonucleotides (5-8,11). The linkage isomerization reaction depends, among other parameters, on the nature of the oligonucleotide phosphodiester backbones and the nucleotide sequence (6,9,11). Not only do nucleotides in the vicinity of the adduct modulate the reactionkinetics, but they may also favor one final product over another. Within DNA.DNA duplexes, the platinum(II) acceptor ligand can be a C-N3 or A-N1 atom (6,12); in the case of a d(GTG).d(CAC) central sequence, both G-N7/C-N3 and G-N7/A-N1 interstrand cross-links are formed, albeit to a different extent (6). On the other hand, only a G-N7/A-N1 cross-link is formed within the corresponding DNA.RNA hybrid. These data suggested that bond strain in 1,3-(G,G)-intrastrand cross-links, the respective orientation of the reactants (platinum moiety and C-N3 or A-N1 nucleophile) within duplexes as well as conformational sampling of the platinated double helices may be important contributors to the rearrangement reaction.

We have extended our previous studies on the nucleotide sequence as a kinetic factor in the transplatin intrastrand cross-link rearrangement reaction. Oligonucleotides containing a transplatin 1,3-(G,G)-intrastrand cross-link were annealed with complementary strands and incubated at 37°C. Aliquots were removed at various times. Formation of the interstrand cross-link was monitored by electrophoresis on a denaturing 24% polyacrylamide gel (figure 2). The results are summarized in table 1. The major kinetic effects can be attributed to the nature of the nucleophile (A-N1 or C-N3), to its environment and location with respect to the platinum(II) moiety and, possibly, to the presence of C residues complementary to the chelated G residues (6,7; table 1).

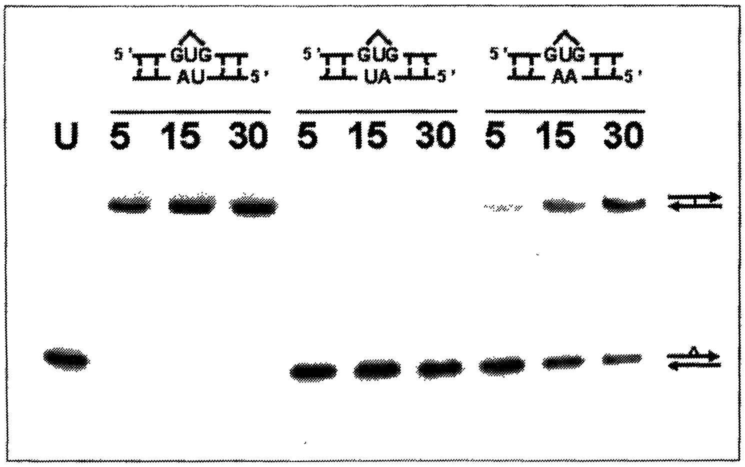

**Figure 2:** Conversion of 1,3-(G,G)-intrastrand adducts into interstrand cross-links within double-stranded oligonucleotides. A representative gel is shown. The oligo(2'-O-Allylribonucleotide) 5'-CUCCUCU**G**U**G**UCUCCUU-3' containing a single 1,3-(G,G)-intrastrand cross-link (chelated residues are in bold font) was paired with the 5'-AAGGAGA[ ]AGAGGAGA-3' RNA strands (where [ ] corresponds to the the AU, UA or AA doublet as indicated above the corresponding lanes). At various times (indicated in minutes above each lane) during incubation at 37°C in 150 mM $NaClO_4$, 5mM phosphate buffer and 0.2mM EDTA, pH 7.5, aliquots were removed and analyzed on a denaturing 24% polyacrylamide gel. The lane U refers to the 5'-end labeled single-stranded 5'-AAGGAGAUAAGAGGAGA-3' oligoribonucleotide.

**Table 1:** Half-lives (in hours) of the 1,3-(G,G)-transplatin intrastrand cross-links within DNA.DNA duplexes

| Platinated strand[a] (5'→3') | Complementary strand[b] (5'→3') | | | | | | | | |
|---|---|---|---|---|---|---|---|---|---|
| | TCTCT | ACTCA | ACTCC | ACACC | ACACA | ATATA | ACATA | AATCA | ACTTA |
| TGTGT | - | 3 | - | - | 12[c] | 15 | > 18 | 8 | 14 |
| AGAGA | 1.6[c] | - | - | - | - | - | - | - | - |
| GGTGT | - | - | 1.2[c] | 4[c] | - | - | - | - | - |
| TG($c_3$)GT | - | 1.5[c] | - | - | 8 | - | - | - | - |
| TGAGT | - | 6[c] | - | - | 24[c] | - | - | | - |

[a] Platinated strands were 20-mer of identical sequence except for the 5 central nucleotides as indicated in the table. $c_3$ represents a propylene moiety replacing the central A or T residue. Platinated G residues are in bold.
[b] The five central nucleotides of the 20-nucleotide long complementary strands facing the 1,3-(G,G)-intrastrand cross-link are shown.
[c] From ref. (6,7)

In a search for factors or conditions that may further accelerate the linkage isomerization and thus make platinated oligonucleotides even more versatile tools for applications in biology (8,11), we have studied the kinetic influence of the platinum(II) inert ligands. Oligonucleotides were reacted with transplatin or platinum(II) complexes (1) to (4) (figure 1) to form the respective 1,3-(G,G)-intrastrand cross-links at the unique GTG site. The linkage isomerization reaction within the d(TGTGT).d(ACACA) sequence was monitored as described above. Results are given in table 2.

Replacement of the transplatin moiety by any of the derivatives (1) to (4) has a dramatic effect on the rearrangement reaction(table 2). While compound (2) slows down the reaction by at least a factor 4, the other derivatives increase the rate of rearrangement 6 to 20 times, the most efficient being compound (1). The derivative (2) has two bulky ligands which may impair proper approach and orientation of reactants along the reaction pathway. Similar observations were made with other platinum(II) complexes having sterically demanding inert ligands such as pyridines or imino-ethers (data not shown). It is interesting to note, however, that compounds (1), (3) and (4) also have bulkier inert ligands than transplatin but still favor the linkage isomerization reaction. Thus, the platinated duplex is able to accommodate these ligands within a productive conformation. In this respect, the flexibility of the inert ligands (as for the linear alkylamines of compounds (1), (3) and (4)) may be an important factor.

**Table 2:** Rearrangement of 1,3-(G,G)-intrastrand cross-links of various transplatin derivatives into interstrand cross-links within 20-mer DNA.DNA duplexes of central sequence d(TGTGT).d(ACACA)

| | Cross-linking agent | | | | |
|---|---|---|---|---|---|
| | transplatin | (1) | (2) | (3) | (4) |
| Half-life (h) | 12[a] | 0.55 | > 50 | 1 | 2 |

[a] From ref. (6)

Compound (1) appeared the most efficient transplatin derivative. To further evaluate its positive effect on the rearrangement kinetics, several other platinated oligonucleotides were prepared. The reactivities of the 1,3-(G,G)-intrastrand cross-links within DNA.DNA duplexes were then evaluated and compared for transplatin and compound (1) (Tables 3 and 4).

**Table 3:** Half-lives (in hours) of 1,3-intrastrand cross-links of transplatin or complex (1) within DNA.DNA duplexes

| Complementary strand[b] (5'→3') | Platinated strand[a] (5'→3') TGAGT | | TGTGT | | TG ($c_3$)GT | | TGAIT | |
|---|---|---|---|---|---|---|---|---|
| | transplatin | (1) | transplatin | (1) | transplatin | (1) | transplatin | (1) |
| ACACA | 24[c] | - | 12[c] | 0.55 | 8 | 0.5 | - | - |
| ACTCA | 6[c] | 0.3 | 3 | 0.25 | 1.5 | 0.15 | 20 | 1 |

[a] Platinated strands were 20-mer of identical sequence except for the 5 central nucleotides as indicated in the table. $c_3$ represents a propylene moiety. Platinated G residues are shown in bold.

[b] The five central nucleotides of the 20-nucleotide long complementary strands facing the 1,3-intrastrand cross-links are shown.

[c] From ref. (6)

**Table 4:** Rearrangement of 1,3-intrastrand adducts of complex (1) into interstrand cross-links within DNA.DNA duplexes

| | Platinated strand (5'→3')[a] | | | | | | | |
|---|---|---|---|---|---|---|---|---|
| | TGTGT | TGTIT | TGAGT | TGAIT | TITIT | TG ($c_3$)GT | TG ($c_2$) IT | TG($c_4$) IT |
| Half-life | 0.25 | 1.25 | 0.3 | 1 | 1.25 | 0.15 | 2.5 | 2.5 |

[a] Platinated strands were 20-mer of identical sequences except for the 5 central nucleotides as indicated in the table. $c_2$, $c_3$ and $c_4$ represent ethylene, propylene and butylene residues, respectively. Platinated G or I residues are shown in bold. In all cases, the complementary strand contained the central d(ACTCA) sequence facing the cross-link.

Within all tested sequences, replacement of the transplatin moiety by derivative (1) increases the reaction rate 10 to 20 fold. In several instances, the half-life of the 1,3-intrastrand adduct is only a few minutes at 37°C. The kinetic effect of the residues in the vicinity of the platinum(II) moiety appears reduced in the case of compound (1) as compared to transplatin. One exception to this rule concerns the platinated 3'-G residue: when it is replaced by an inosine, the linkage isomerization reaction is significantly slowed down (table 4). This result reveals that the 3'-G-$NH_2$ moiety is important for the reaction kinetics; it is likely involved in hydrogen-bonding with the complementary C residue, whose replacement by a T residue also slows the reaction (table 1). Such interactions could contribute primarily to the positioning of the reactants rather than to a large increase of ring strain. Indeed, replacement of the central N residue within a GNI sequence by an ethylene or butylene moiety does not havea dramatic effect on the reaction (table 4).

Finally, we studied the rearrangement of 1,3-(G,G)-intrastrand cross-links of complex (1) into interstrand cross-links within (2'-O-Methyl-RNA).RNA hybrids. In the case of transplatin, the reaction can be extremely fast within certain sequences and we have successfully tested platinated oligo(2'-O-Methyl-ribonucleotide)s as antisense agents in *in vitro* and *ex vivo* assays (8,11). Considering our previous data (8), a significant acceleration of the linkage isomerization reaction for a broader range of target sequences could represent an important improvement for antisense applications. However, replacement of transplatin by derivative (1) within the platinated (2'-O-Methyl-RNA).RNA hybrids has very little effect on the slower linkage isomerization reactions (table 5). Only within the (2'-O-Methyl-r(UGUGU)).r(AUGA) hybrid is the rearrangement reaction 10-times faster.

**Table 5:** Half-lives (in hours) of the 1,3-(G,G)-intrastrand cross-links of transplatin or complex (1) within (2'-O-Methyl-RNA).RNA hybrids

| | complementary strand (5'→3')[a] | | | | | | | |
|---|---|---|---|---|---|---|---|---|
| | ACACA | ACAUA | AUGCA | AUCCA | AGAA | AAAA | AAUA | AUGA |
| transplatin | 3[b] | 4[b] | 1 | 0.5 | 0.5[b] | 0.5[b] | > 24[b] | > 24 |
| (1) | 2 | 4 | 0.6 | 0.3 | 2 | 1 | > 24 | 2.25 |

[a] In all cases, the platinated strand (top strand) was a 18-mer oligo(2'-O-Methyl-ribonucleotide) containing the UGUGU central sequence.
[b] From ref.(8)

### DISCUSSION

Within platinated double-stranded oligodeoxyribonucleotides containing the d(GNG).d(CN'C) sequence (N and N' are A, T or C), 1,3-intrastrand adducts rearrange into thermodynamically stable interstrand cross-links (6,7). These interstrand cross-links are formed between the 5'-G of the GNG motif and its complementary C residue. When N' = A, a second minor cross-link is also formed between the 5'-G and the A residue. Within DNA.DNA duplexes, the linkage isomerization reaction occurs within all the sequences that were studied but the reaction rates can be dramatically different ($1 < \tau_{1/2} < 50h$, at 37°C). Not all residues in the vicinity of the adduct are of equivalent influence. For instance, the base and sugar of the central N residue have little effect on the kinetics and N can even be replaced by a propylene moiety (table 1; ref. (7)). By contrast, the nature of the opposite N' residue can significantly alter the reaction rate (table 1). The rearrangement is about five times slower when N' is a purine as compared to a pyrimidine. A purine on the 5'-edge of the intrastrand cross-link (as in the GGTGT sequence) also favors reaction kinetics (table 1). Another parameter that can greatly interfere with the rearrangement reaction is the nature of the platinum(II) inert ligands. Significant variations of theintrastrand cross-link half-lives (up to two orders of magnitude) were observed for various bulky inert ligands (table 2); substitution of the $NH_3$ ligands of transplatin by linear alkylamines can lead to linkage isomerization reactions that are complete in less than one hour at 37°C. Although all tested transplatin derivatives have two identical inert ligands (figure 1), it could be of interest to assess the effect of asymetric platinum(II) complexes on the linkage isomerization reaction.

The local oligonucleotide sequence at the site of the 1,3-cross-link can also drastically change the reaction rate within platinated DNA.RNA or (2'-O-R-RNA).RNA hybrids (R being a methyl or allyl group). However, in such hybrids, the maximal effects coincide with the deletion of one of the residues facing the intrastrand cross-link (figure 2; ref.(8)). The permutation of the nucleotides facing the intrastrand cross-link can also have a strong effect on the reaction (figure 2; (8)). On the other hand, replacement of the transplatin moiety by derivative (1) only marginally improves the rearrangement kinetics within platinated (2'-O-R-RNA).RNA hybrids (table 5). Finally, it is to be noted that, in the final product, the cross-linked atom within the RNA complementary strand is predominantly a A-N1 rather than a C-N3 (8).

In order to rationalize these data, one should assume that the rearrangement reaction proceeds through direct nucleophilic attack (SN2 mechanism; ref. (9)) and should consider the following scheme:

$$A(Pt) + B \underset{}{\overset{(1)}{\rightleftharpoons}} A(Pt).B \underset{}{\overset{(2)}{\rightleftharpoons}} [A(Pt).B]^{*} \overset{(3)}{\longrightarrow} A\text{-}Pt\text{-}B$$

where A(Pt) represents the top strand containing a 1,3-intrastrand adduct, B is the bottom strand, A(Pt).B is the platinated duplex in a non-productive or productive (*) conformation and A-Pt-B is the duplex containing an interstrand cross-link.

The 1,3-intrastrand adduct is usually stable within the single-stranded A(Pt) species. The formation of the A(Pt).B double helix (step 1), which brings the reactants in close proximity, is therefore critical to the rearrangement reaction. As is the case for theassembly of *trans*-acting ribozymes, the limitation for step 1 is likely the rate of diffusion of oligonucleotides within aqueous media, close to $10^8$ $M^{-1}.s^{-1}$at 37°C (13). If step 1 was the rate-limiting step of the linkage isomerization reaction, the half-life of the intrastrand cross-link within the duplex would be about $10^{-4}$ s (for micromolar solutions of oligonucleotides), several orders of magnitude lower than our experimental values (see tables 1-5). Thus, the rate-limiting step for therearrangement reaction is probably subsequent to the annealing of both strands. All collected data suggest that an important factor for the reaction is the respective orientation of reactants rather than a mere increase in their local effective concentrations. However, the optimal set-up of reactants is not necessarily obtained right after pairing (step 1) and a potentially rate-limiting conformational change (step 2) may be required to properly align the reactive entities. Therefore, conformational sampling of the platinated duplex or hybrid could also be a major kinetic factor. In the rigid A-form helices adopted by (2'-O-R-RNA).RNA hybrids (14), reactants would have to be ideally positioned right after pairing for the reaction to proceed quickly and efficiently. This is likely the case for the platinated (2'-O-R-r(UGUGU)).r(AUAA) hybrids in which the isomerization is extremely fast (figure 2; ref. (8)) and the chemical step (step 3) is potentially rate-limiting. Within suchrigid hybrids, substitution of the platinum(II) inert $NH_3$ ligands by bulkier but still flexible alkylamines should have only limited consequences on conformation and therefore little kinetic effect (table 5; ref. (11)). By contrast, permutation of the two residues facing the intrastrand cross-link displaces the RNA nucleophile a few angstroms away from the platinum(II) center, which dramatically slows down the rearrangement reaction (figure 2; ref. (8)). It seems reasonnableto assume that the rigid hybrid cannot easily adjust to the new set-up of reactants. The situation appears different for DNA.DNA duplexes, whose flexibility allows various nucleophiles within the complementary strand to react with the platinum(II) centerto form interstrand cross-links (table 1; ref. (6)). It is tempting to speculate that, in the case of DNA.DNA duplexes, the acceleration of the linkage isomerization reaction upon replacement of the transplatin moiety by derivative (1), (3) or (4) is due to ligand-induced sterical reduction of conformational sampling and locking of the duplex central region into conformations that insure a productive positioning of reactants.

Another factor potentially important for the linkage isomerization reaction is bond strain within the 1,3-intrastrand cross-link. Although it is difficult to experimentally weigh its role against those of other parameters, several lines of evidence suggest that bond strain may be a non-negligible driving force of the isomerization reaction. For instance, within single-stranded oligonucleotides containing a d(CGNG) sequence, 1,3-(G,G)-intrastrand cross-links slowly evolve into less constrained, thermodynamically favored, 1,4-(C,G)-intrastrand adducts (4,5). Moreover, the Pt-G bond that is broken during the linkage isomerization reactions depends on whether the oligonucleotides are single- or double-stranded (5,6). It is possible that restriction of conformational freedom resulting from annealing with the complementary strand and/or from the approach of the nucleophile transfers a large part of the strain due to the platinum(II) chelation and to sterical crowding into a single chemical bond concomitantly to the productive alignment of reactants. This would result in lowering the activation energy barrier to the linkage isomerization reactions in a mechanism similar to enzymatic reactions (15-17).

Oligonucleotides containing 1,3-intrastrand adducts which spontaneously and specifically cross-link complementary RNA or DNA targets can be useful molecular tools in biology and medicine. In fact, we have previously demonstrated that platinated oligo(2'-O-methylribonucleotide)s are efficient antisense agents that specifically inhibit the expression of their target gene, *in vitro* or in cultured cells (8,11). A key factor to the successful use of platinated oligonucleotides is the rate of the rearrangement reaction. Within complex biological media, a fast cross-linking reaction is required so that the oligonucleotide has a high probability to covalently associate with its target during their rare bimolecular uncounters and before cellular machineries such as highly processive polymerases trigger their dissociation. In this paper, we present data that should facilitate the design of platinated oligonucleotides that quickly and efficiently cross-link a broader array of

RNA and DNA targets. By manipulating the nature of the platinum(II) ligands, the sequence and the backbone chemistry of platinated oligonucleotides, it should be possible to obtain fast rearrangement reactions within a variety of sequences. Adapting the SELEX method (3) to this task could result in a cost-effective, high-throughput screening of newand specific platinum(II)-based drugs. Then, platinated oligonucleotides that efficiently cross-link to non nucleic acid targets, such as cellular proteins, may also be evolved from large pools of randomized molecules.

### ACKNOWLEDGMENTS
We are grateful to Drs Rachid Rahmouni, Tran Thanh Thu and Annie-Claude Albert for critical reading of the manuscript. This paper is dedicated to the memory of our mentor Marc Leng who deceased on May 7th, 2000.

### REFERENCES
1. Pyle, A. M. (1993) *Science* **261,** 709-14.
2. Pyle, A. M. (1996) *Met. Ions Biol. Syst.* **32,** 479-520.
3. Williams, K. P. & Bartel, D. P. (1996) in *Catalytic RNA*, eds. Eckstein, F. & Lilley, D. M. J. (Springer-Verlag, Berlin-Heidelberg), **Vol. 10**, pp. 367-81.
4. Comess, K. M., Costello, C. E. & Lippard, S. (1990) *Biochemistry* **29,** 2102-10.
5. Dalbies, R., Boudvillain, M. & Leng, M. (1995) *Nucleic Acids Res.* **23,** 949-53.
6. Dalbies, R., Payet, D. & Leng, M. (1994) *Proc. Natl. Acad. Sci. U S A.* **91,** 8147-51.
7. Boudvillain, M., Dalbies, R., Aussourd, C. & Leng, M. (1995) *Nucleic Acids Res.* **23,** 2381-8.
8. Boudvillain, M., Guerin, M., Dalbies, R., Saison-Behmoaras, T. & Leng, M. (1997) *Biochemistry* **36,** 2925-31.
9. Boudvillain, M., Dalbies, R. & Leng, M. (1996) *Met. Ions Biol. Syst.* **33,** 87-104.
10. Distefano, M. D., Shin, J. A. & Dervan, P. B. (1991) *J. Am. Chem. Soc.* **113,** 5901-02.
11. Colombier, C., Boudvillain, M. & Leng, M. (1997) *Antisense Nucleic Acid Drug Dev.* **7,** 397-402.
12. Andersen, B., Bernal-Méndez, E., Leng, M. & Sletten, E. (2000) *Eur. J. Inorg. Chem.* **6,** 1201-10.
13. Cech, T. R., Herschlag, D., Piccirilli, J. A. & Pyle, A. M. (1992) *J. Biol. Chem.* **267,** 17479-82.
14. Cummins, L. L., Owens, S. R., Risen, L. M., Lesnik, E. A., Freier, S. M., McGee, D., Guinosso, C. J. & Cook, P. D. (1995) *Nucleic Acids Res.* **23,** 2019-24.
15. Sella, A., Basch, H. & Hoz, S. (1996) *J. Am. Chem. Soc.* **118,** 416-20.
16. Mesecar, A. D., Stoddard, B. L. & Koshland, D. E. J. (1997) *Science* **277,** 202-6.
17. Narlikar, G. J. & Herschlag, D. (1998) *Biochemistry* **37,** 9902-11.

